# Renal Disease and Kidney Transplantation in Hispanic American Persons

**DOI:** 10.34067/KID.0000000579

**Published:** 2024-09-16

**Authors:** Girish K. Mour, Aleksandra Kukla, Andres Jaramillo, Daniel S. Ramon, Hani M. Wadei, Mark D. Stegall

**Affiliations:** 1 Division of Nephrology, Mayo Clinic, Phoenix, Arizona; 2 Division of Nephrology and Hypertension, Mayo Clinic, and William J. von Liebig Center for Transplantation and Clinical Regeneration, Mayo Clinic, Rochester, Minnesota; 3 Department of Laboratory Medicine and Pathology, Mayo Clinic, Scottsdale, Arizona; 4 Department Pathology, Microbiology and Immunology, Vanderbilt University Medical Center, Nashville, Tennessee; 5 Department of Transplant Medicine, Mayo Clinic, Jacksonville, Florida; 6 Division of Transplantation Surgery, Department of Immunology, Mayo Clinic, and William J. von Liebig Center for Transplantation and Clinical Regeneration, Mayo Clinic, Rochester, Minnesota

**Keywords:** chronic renal disease, ESKD, ethnic minority, human genetics, kidney transplantation, minority health and disparities

## Abstract

The Hispanic population of the United States is the second largest racial or ethnic group, comprising 18.7% of the population. However, this population is incredibly heterogeneous differing in genetic traits, cultural upbringing, educational backgrounds, and financial status. The impact of this heterogeneity on the prevalence and outcomes of renal disease and kidney transplantation is understudied compared with non-Hispanic White and Black populations. What is known appears to be underrecognized. This review aims to critically assess current medical literature on Hispanic individuals, focusing on etiological factors, disease progression, and outcomes related to CKD and kidney transplantation. By doing so, we aim to underscore key areas for further in-depth investigation.

## Introduction

The US Census officially recognizes two different ethnicities: Hispanic or Latino and not Hispanic or Latino.^1^ In 2020, 18.7% of the population (more than 61 million people) identified as Hispanic or Latino.^1–3^ Of them, approximately 62% identified as Mexican ethnicity. Taken separately, the US Hispanic population would rank 23 among the largest countries (after France with 65.2 million), and the Mexican American population equals the population of Canada (37 million).^2^ A deeper understanding of the complex diversity of Hispanic persons can help to develop preventive strategies and targets for interventions and, consequently, improve health care of this population. The present review sought to highlight the complexity of the Hispanic population in terms of risks and outcomes of kidney disease and transplant and to identify critical areas for future research.

## Origins of US Hispanic Populations

Ancient and recent migrations in the Western Hemisphere contribute significantly to the genetic diversity of the Hispanic population in the United States. Many Mexican American individuals share strong genetic ties with Native American individuals, both descended primarily from Paleoamerican population who migrated from Asia around 11,000 years ago. Diverse indigenous groups in Mesoamerica developed distinct cultures and genetics. The arrival of European population in the late 15th century, along with African and Asian slaves, led to the creation of mixed-race mestizos. ^4^

**Figure 1:**
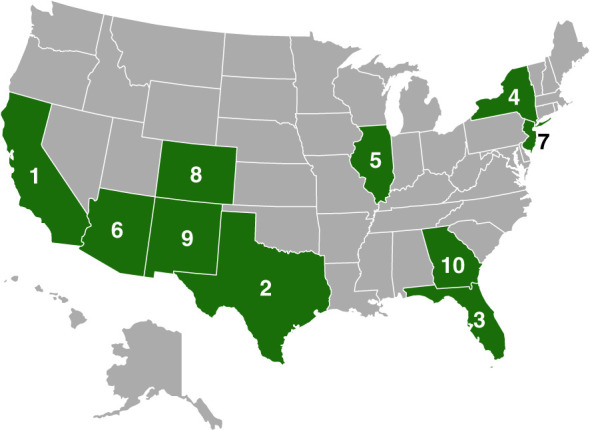
**Map showing the ten US states with the largest Hispanic populations.** (From ref. 5).

**Figure 2:**
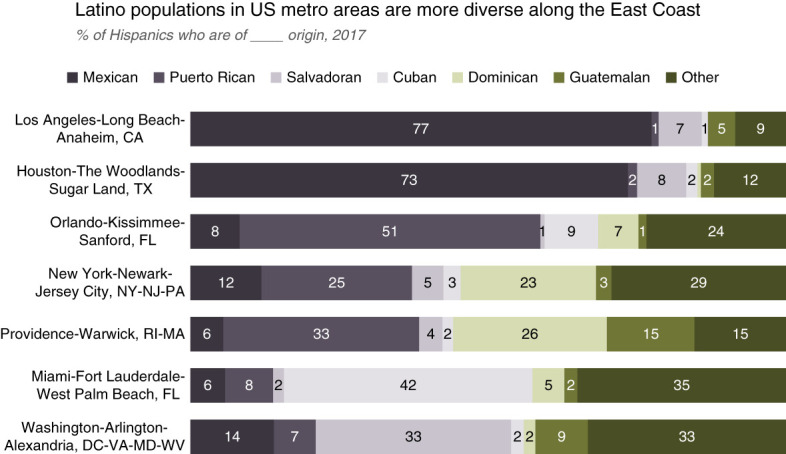
**Hispanic groups in different US metropolitan areas, 2017.** This figure shows that the country of origin of Hispanic persons in different US cities differs widely. For example, Hispanic individuals in the Los Angeles and Houston areas primarily identify Mexico as their country of origin, whereas the majority in Orlando identify Guatemala as their country of origin. Other cities have a mixture of populations with no clear majority. The subgroups are more diverse in the Eastern United States (From ref. 8). CA, California; DC, District of Columbia; FL, Florida; MA, Massachusetts; MD, Maryland; NJ, New Jersey; NY, New York; PA, Pennsylvania; RI, Rhode Island; TX, Texas; VA, Virginia; WV, West Virginia.

In 2019, California, Arizona, Texas, and New Mexico accounted for almost 50% of Hispanic persons in the United States (Figure 1), most of whom are Mexican American persons.^6^ By contrast, Hispanic persons in the Eastern United States more commonly trace their origins from the Caribbean, including Puerto Rico, the Dominican Republic, and Cuba^7^ (Figure 2).

## Genetic Heterogeneity

Genetic studies confirm that Hispanic populations are genetically diverse, with variations across geographic regions (Figure 2).^7^ Genetic ancestry patterns vary significantly among Hispanic individuals. A study of 142 Latino individuals found that the US cohort had 48% European and 40% American Indian ancestry, the Mexico cohort had slightly higher similar proportions, and Brazil and Colombia had 70% European ancestry.^9^ Within the Hispanic population, Mexican persons had more American Indian ancestry, whereas Puerto Rican and Dominican persons had primarily European and African ancestry,^10^ reflecting migration patterns, local admixing, and the geographic area of residence.

## Cultural and Socioeconomic Factors

Disparities in health insurance, income levels, and educational attainment contribute to increased risks of various diseases in Hispanic subgroups. Addressing these underlying differences is crucial for developing effective disease management strategies and reducing health care disparities in the Hispanic population.

The lack of granular Hispanic population data and the tendency to aggregate all Hispanic individuals into one group are common. In 2019, 19% of Hispanic persons were uninsured, in contrast to 8% of non-Hispanic White persons.^11^ Of uninsured Hispanic persons, 30% were in the age group of 18–64 years compared with 10.5% of White persons in a similar age group.^12^ Among Hispanic subgroups, 20% of Mexican, 8% of Puerto Rican, 14% of Cuban, and 29% of Central American individuals lacked health insurance. Hispanic median household income was $14,000 less than non-Hispanic White persons, with lower educational attainment and 28% lacking English fluency.^11^

These factors heighten the risk of major diseases and limit access to proper disease management. Financial constraints hinder access to a nutritious diet, a combination of American and Hispanic foods rather than Hispanic foods alone,^13^ increasing obesity and diabetes mellitus (DM) risk. Poor access to health care and inability to navigate the health care system contribute to poor outcomes in diabetes^14,15^ and CKD.^16–18^

## The Hispanic Paradox

Despite the challenges to receiving health care faced by large numbers of Hispanic persons, overall life expectancy is higher than non-Hispanic White persons.^19^ The reasons for this Hispanic paradox are unclear, but it appears to mainly due to improved survival in older people, particularly Mexican American persons. Death rates in young and middle-aged Hispanic persons are similar to non-Hispanic White persons. Potential explanations include better family support for older individuals, acculturation,^20^ low prevalence of smoking (7.7% in Hispanic versus 13% in Non-Hispanic White persons),^21^ mortality underreporting, and reverse migration back to home country at later stages of life.

## DM

Type 2 DM is the leading cause of renal failure in the United States and the most common indication for kidney transplant. Approximately 34 million American persons (10.5%) have DM,^22^ and the prevalence of DM is increasing annually.^23^ American Indian or Alaskan Native persons have the highest diabetes prevalence (14.7%), followed by Hispanic persons (12.5%). Amongst Hispanics, Mexican American persons have the highest prevalence (14.4%), followed by Puerto Rican descendants. Prediabetes also has a high prevalence among Hispanic persons. The National Health and Nutrition Examination Survey data from 2011 through 2016^24^ showed a 41% prevalence of prediabetes.

In the Hispanic Community Health Study/Study of Latinos, 37% of diabetic cases were undiagnosed DM.^25^ Factors associated with undiagnosed DM included Cuban and South American ethnicity, female sex, lack of family history, and lack of health insurance. Disparities exists in albuminuria prevalence,^26^ with Mexican American individuals having low rates than Black individuals with or without DM. When adjusted for multiple factors, including age, body mass index, hemoglobin A_1c_ value, and hypertension, Mexican American persons without DM did not have greater odds of albuminuria than White persons without DM.

Research from the Multi-Ethnic Study of Atherosclerosis^27^ suggests that European ancestry may be protective against albuminuria in Puerto Rican persons, although this effect diminished after adjustments. The results show that although ancestry may determine albuminuria prevalence, other determinants of health care may supersede ancestry. Hispanic persons are more likely to have poorer glycemic control at DM diagnosis and follow-up compared with non-Hispanic White individuals.^28^ Similarly, sodium-glucose cotransporter-2 inhibitors, glucagon-like peptide-1 agonists, and weight loss surgeries were less likely to be prescribed in Hispanic persons,^29,30^ despite efficacy of such therapies in Hispanic persons.^31,32^

## Obesity

The age-adjusted prevalence of obesity among Hispanic persons is 45%, compared with 50% among non-Hispanic Black and 42% among non-Hispanic White persons.^33^ Social determinants and environmental exposure, such as being US born versus non-US born, contribute to these higher obesity rates.^34^ However, geographic differences exist within the US Hispanic population. Hispanic persons living in Bronx, New York, were more likely to have obesity than Hispanic persons in Chicago, Miami, and San Diego.^13^ Low socioeconomic conditions—especially education level, low household income, and barriers to physical activity—play an important role in obesity outcomes.^35,36^

As stated above, dietary patterns may predispose to obesity, but dietary intervention alone may not produce long-term solutions. A culturally based intervention and modern technology involving Latino population achieved a weight loss of at least 5% at 12 months, but the benefits diminished by 24 months, underscoring the need for sustained approaches to weight management.^37^ Unfortunately, the penetration glucagon-like peptide-1 agonist and weight loss surgery remain low in Hispanic persons.^30,38^

## CKD

Fifteen percent of the US population has CKD, with Hispanic persons accounting for 14% of cases.^39^ Data from the United States Renal Data System^40^ cites a CKD prevalence of 12.5% in Mexican American persons. Within Hispanic persons, differences exist in CKD prevalence, as reported by the Hispanic Community Health Study/Study of Latinos,^41–45^ where 40% of the population were Mexican American persons. The incidence of CKD was the highest in the Puerto Rican persons (15/1000 person-years), followed in frequency by Mexican American persons (10/1000 person-years).^45^ The overall prevalence of CKD in Hispanic persons is similar to non-Hispanic White persons.

The term acculturation relates to the population act of absorbing local cultures and customs and especially applies in immigrant population. Lower language acculturation subscores were associated with a 30% increase in CKD prevalence among patients older than 65 years.^43^ This is particularly significant given that CKD risk rises with age, and comorbid conditions heighten susceptibility. The added language barriers may exacerbate CKD progression in older adults, limiting their access to health care and understanding of complex treatments. Intervention programs targeting the older high-risk group, supported by Hispanic navigators, may mitigate this issue. The complex interplay between acculturation, socioeconomic status, and education level is evident in two studies, prompting speculation about the potential genetic contribution to CKD within the Hispanic population.^43,45^

Sex differences further complicates CKD in Hispanic persons.^46^ CKD prevalence in Hispanic subgroups is highest among Puerto Rican women, followed by Mexican American women, whereas the prevalence among men in these groups is nearly identical. However, multivariable analysis suggests that CKD risk was similar across different Hispanic backgrounds, indicating that factors beyond ethnicity, such as socioeconomic status, access to health care, lower household income, lower education level, and lack of insurance, play significant roles.^16,47^

The CKD progression to ESKD also varies with race and ethnicity. In the Chronic Renal Insufficiency Cohort study,^46^ CKD progression and incident ESKD rates were significantly higher in Hispanic patients than in non-Hispanic White patients (2.6 versus 1.4 per 100 person-years). Kaiser Permanente Renal Registry^48^ highlights a two-fold increase in the age- and socioeconomic-adjusted ESKD rate in the Hispanic population. A DM diagnosis and age 50 years or older increased the ESKD risk. These findings underscore the influential roles of diabetes, proteinuria, and factors like acculturation, socioeconomic status, and education level in CKD progression, potentially outweighing the impact of ethnicity alone.

An additional major barrier is the lack of a validated questionnaire to quantify these issues in the different Hispanic subgroups. Grouping college-educated, affluent Hispanic persons with predominantly European ancestry with Hispanic persons who are poor, not fluent in English, with predominantly American Indian or Black ancestry creates barriers to identifying of high-risk groups, complicating targeted interventions.

## ESKD

From 2000 to 2016, the standardized ESKD incidence rate among Hispanic persons decreased (from 650/million years to 450/million years), but was still slightly higher than that of non-Hispanic White persons (stable at 350 million years during that time span).^49^ Conversely, the standardized prevalence of ESKD increased at similar rates in both groups, with Hispanic persons experiencing higher prevalence (3000 per million person-years) compared with non-Hispanic White persons (1940 per million person-years). The major factor leading to the increased overall prevalence is the increasing survival of patients with ESKD—both on dialysis and with kidney transplantation.

The Hispanic population has lower rates of dialysis withdrawal compared with the non-Hispanic White population. The Hispanic population also has a lower 1-year death rate per 100 patient-years at risk^50^ than the non-Hispanic White population. However, when examining Hispanic subgroups, distinctions emerge. Mexican American persons have the lowest transplant rates and 1-year unadjusted death rate per 100 patient-years at risk, but higher withdrawal rates than Cuban or Puerto Rican individuals.^50^ Mexican persons in the United States had a significantly lower adjusted mortality rate—by 21%—than non-Hispanic White persons. In the United States, Puerto Rican persons had a 2-year adjusted relative mortality risk that was 30% higher than Mexican American persons, whereas no differences were seen between Cuban and Mexican American persons.

In an incident ESKD population,^19^ Hispanic patients had a lower mortality rate than the non-Hispanic population (53% versus 68%). This difference in mortality rates was significant at a younger age (33% reduced mortality rate in ages 18–59 years) compared with non-Hispanic White individuals. Even when considering kidney transplant as a competing event, Hispanic patients maintain a lower mortality rate across various age groups. Taken together, these outcomes could imply that the progression of kidney disease may be faster in the Hispanic population, leading to ESKD with less cardiovascular disease.

## Kidney Transplantation

Hispanic persons represent the third largest racial/ethnic group on the United Network for Organ Sharing deceased donor kidney waiting list (47% non-Hispanic White, 30% Black, and 21% Hispanics).^51^ In 2019, only 19% of the Hispanic persons received an organ transplant compared with 45% of non-Hispanic White persons, while contributing significantly less to both deceased and living donor pool.^52^ Access to kidney transplantation in Hispanic persons could be related to misunderstanding and mistrust toward kidney transplantation,^53^ lack of information regarding transplantation from providers,^54^ bias in evaluation, waitlisting, and transplantation.^55^ Contrastingly, once a transplant evaluation has started, Hispanic persons are more likely to complete evaluation as compared with Black persons,^56^ although rates of approval and listing remain lower than non-Hispanic White persons.^55^

The discrepancy in deceased donor kidney transplant diminishes when accounting for competing events, such as mortality in non-Hispanic White persons, blood type, and the preferential residence of Hispanic individuals in organ procurement organization (OPO) regions with longer wait times (see Figure 1).^57^ Notably, when adjusting for the OPO, Hispanic patients exhibited higher transplant rates than non-Hispanic White patients, underscoring the influence of listing OPO on wait times among minority populations.^58^

Disparities extend to waitlist status changes.^59,60^ Non-Hispanic White patients were more likely to be reactivated on waitlist compared with Hispanic and Black populations. The inability to get reactivated on waitlist among the Hispanic patients could be due to unresolved medical issues, financial constraints, lack of social support, and challenges to complete transplant-related workups. Importantly, the likelihood of receiving a living donor kidney transplant is lower in Hispanic than non-Hispanic White patients.^61^ The latter could be due to opposition from family members because of concerns for being living donors,^62^ access to health care and socioeconomic factors,^63^ knowledge lack, cultural beliefs, and potential impact on health after living donation.^62^

Similarly, Hispanic persons have a lower odds ratio of receiving preemptive kidney transplant in post-kidney allocation system (KAS) changes than pre-KAS changes compared with White patients,^64^ although overall monthly transplant rates increased from 0.79% to 0.91% in Hispanic patients post-KAS.^65^ Medicare coverage, a lower education completion level, younger age, and male sex were characteristics associated with a lower likelihood of a preemptive kidney transplant. However, some of these differences in preemptive transplantation has been overcome by expansion of Medicaid under Affordable Care Act.^66^ Arce *et al.* analyzed ^67^ the United States Renal Data System transplant data and found (*1*) Hispanic White patients had a lower risk of mortality and all-cause graft loss compared with non-Hispanic White patients; (*2*) the risk of graft failure, excluding death with a functioning graft, was lower in Hispanic patients older than 60 years (possibly associated with life-long Medicare coverage as age increases). Importantly, graft losses not due to death were similar in Hispanic and non-Hispanic White patients in recipients <60 years. Similarly, a recent Scientific Registry of Transplant Recipients report also suggests that Hispanic White recipients (from 2011) have higher graft and patient survival at 5 years compared with non-Hispanic White recipients.^68^

Table 1 summarizes important studies in the US Hispanic population. Despite being disadvantaged, recent studies suggest that Hispanic kidney transplant recipients have better overall outcomes than non-Hispanic White recipients. These improvements in survival are interesting given the greater number of HLA mismatches, longer pretransplant dialysis and less frequent preemptive kidney transplants, and living donor transplant compared with non-Hispanic White patients. Another possibility is selection bias by transplant programs favoring transplantation of healthier Hispanic persons. It should be remembered that the Hispanic population is very heterogeneous. Issues related to outcomes based on socioeconomic status, fluency in English, and other potential barriers to care would be interesting to investigate in this population to determine the mechanism of improved outcomes.

**Table 1:** Studies of Hispanic patients and outcomes

Authors (Year)	Hispanic Subpopulation	Study Database	Outcome
Ku *et al.*^18^ (2020)	ESKD	USRDS	Less likely to receive a referral and have a lower living donor transplant rate for the first 3 yr of dialysis
Vranic *et al.*^58^ (2014)	ESKD	SRTR	Highest median wait time for receipt of deceased donor transplant, which could be an OPO effect
Purnell *et al.*^61^ (2013)	Living donor transplant	USRDS	Lower living donor transplant rates than White patients
King *et al.*^64^ (2019)	Preemptive transplant	SRTR	Lower odds of preemptive kidney transplantation
Arce *et al.*^67^ (2015)	Survival and allograft loss	USRDS	Better survival and had lower graft loss, including death

OPO, organ procurement organization; SRTR, Scientific Registry of Transplant Recipients; USRDS, United States Renal Data System.

## Improving Access

Access to kidney transplant could be patient related, provider bias, and systemic failures.^69^ Social workers play a crucial role in helping patients navigate the complex transplant process and understand insurance information. Overcoming language barriers is essential, which can be achieved through dedicated Spanish interpreters, Spanish-speaking providers, and involving family members and caregivers in the treatment plan, especially for elderly patients. Providing language-specific materials, community outreach programs, and patient education initiatives are also vital to improving transplant access.^70^

Addressing these factors requires culturally competent programs tailored to individuals' beliefs and customs. Such programs aim to reduce mistrust, overcome language barriers, bridge knowledge gaps in dialysis units, promote workforce diversity, address financial concerns for living donors, and alleviate family concerns.^71–73^ These initiatives can be implemented successfully without significant financial impact.^74^ In addition, eliminating bias in every step of the transplant process, from evaluation to post-transplant care, is crucial to increasing transplant rates. Transplant centers must communicate clearly with referral nephrologists about patients' progress and address issues during the waitlist and post-transplant follow-up care. Referring patients to centers with shorter wait times should also be considered.

Population-level interventions should scrutinize organ allocation policies to ensure equity, particularly in regions with higher minority populations. On a broader scale, mitigating poverty—a significant social determinant—can enhance health literacy, reduce language barriers, improve diet, and increase health care access. This, in turn, can lower the incidence of renal failure and the need for transplantation, ensuring more equitable health care outcomes.

## Potential Areas of Research

A major goal of this review of research on Hispanic persons was to identify patients at elevated risk for poor outcomes and to design more effective intervention strategies. We contend that the Hispanic category is too broad to achieve this goal and needs further refinement.

Specific health questions for race and ethnic groups need investigation (Box 1). For example, obesity and type 2 DM are epidemics in all racial and ethnic groups, but does a single approach work equally in all groups without accounting for socioeconomic status and genetic factors? What is the role of human leukocyte antigen and apolipoprotein L1 in the various Hispanic subgroups? Is the risk of renal failure after being a living donor the same for all Hispanic subgroups? Is the risk of renal failure the same for all Hispanic subgroups when corrected for age, obesity, and DM? Can investigators develop methods to improve the preventive health care in pre-ESKD phase that is lacking generally? In outcome studies, how can investigators adjust for factors such as a language barrier, socioeconomic status, and age to evaluate cultural differences?

Box 1 **Suggested Topics of Future Clinical Trials to Address Major Unmet Needs in Kidney Transplantation**

Creation of a database of Hispanic persons of different subgroups and analysis of outcomes in these subgroups and the role of HLA.

Role of apolipoprotein L1 in Hispanic persons with Black ancestry.

Creation of a questionnaire to assess socioeconomic status in detail.

Evaluate living donation in the different Hispanic subgroups—both rate and outcomes.

Role of minority educated and language-specific patient navigators and success rates in waitlisting and subsequent transplant surgery.

Roles of genetics in disease development, including diabetes and obesity.

Factors affecting rates of rejection, allograft loss, and death in Hispanic recipients after kidney transplantation.

## Conclusion

The US Hispanic population is large, diverse, and changing. It remains a large, understudied cohort that is at extreme risk of ESKD in the next few decades. The need is pressing to perform detailed studies in well-defined cohorts for better understanding of the issues related to disease risk and outcome in Hispanic individuals. By doing more research, we are hopeful that novel interventions can be developed to enhance clinical outcomes in this population.
